# Correction to Acknowledgement

**DOI:** 10.1186/s41205-021-00126-4

**Published:** 2021-11-17

**Authors:** 3D Printing in Medicine

**Affiliations:** London, UK

Following publication of the articles [1, 2], it is noticed the below sentence needs to be added to the Acknowledgement section of the two articles:

The article processing charge for this publication has been funded by an unrestricted grant from Formlabs.

Following publication of the articles [3–8], it is noticed the below sentence needs to be added to the Acknowledgement section of these articles:

The article processing charge for this publication has been funded by an unrestricted grant from Materialise.

The original articles have been updated.
